# Novel PITX2 Mutations including a Mutation Causing an Unusual Ophthalmic Phenotype of Axenfeld-Rieger Syndrome

**DOI:** 10.1155/2019/5642126

**Published:** 2019-07-01

**Authors:** Liqin Huang, Yong Meng, Xiangming Guo

**Affiliations:** ^1^Department of Ophthalmology, Changzhou No. 2 People's Hospital, Changzhou 213000, Jiangsu Province, China; ^2^Department of Ophthalmology, Changzhou No. 3 People's Hospital, Changzhou 213000, Jiangsu Province, China; ^3^State Key Laboratory of Ophthalmology, Zhongshan Ophthalmic Center, Sun Yat-sen University, Guangzhou 510060, China

## Abstract

**Purpose:**

The aims of this study were to examine novel mutations in PITX2 and FOXC1 in Chinese patients with anterior segment dysgenesis (ASD) and to compare the clinical presentations of these mutations with previously reported associated phenotypes.

**Methods:**

Twenty-six unrelated patients with different forms of ASD were enrolled from our paediatric and genetic eye clinic. The ocular manifestations of both eyes of each patient were recorded. Genomic DNA was prepared from venous leukocytes. All coding exons of PITX2 and FOXC1 were amplified by polymerase chain reaction (PCR) from genomic DNA and subjected to direct DNA sequencing. Analysis of mutations in control subjects was performed by heteroduplex single-strand conformation polymorphism (SSCP) analysis.

**Results:**

Sequence analysis of the PITX2 gene revealed four mutations, including c.475_476delCT (P.L159VfsX39), c.64C > T (P.Q22X), c.296delG (P.R99PfsX56), and c.206G > A (P.R69H). The first three mutations were found to be novel. The c.475_476delCT (P.L159VfsX39) mutation, located at the 3′ end of the PITX2-coding region, was identified in a Chinese Axenfeld-Rieger syndrome (ARS) patient who presented with an unusual severe phenotype of bilateral aniridia. The clinical characteristics, including the severity and manifestations of the patient's phenotype, were compared with reported PITX2-associated aniridia phenotypes of ARS in the literature.

**Conclusions:**

These results expand the mutation spectrum of the PITX2 gene in patients with ARS. The PITX2 gene may be responsible for a significant portion of ARS with additional systemic defects in the Chinese population. This is the first reported case of a mutation at the 3′ end of the PITX2-coding region extending the phenotypic consequences to bilateral aniridia. The traits of ARS could display tremendous variability in severity and manifestations due to the dominant-negative effect of PITX2. Our results further emphasize the importance of careful clinical and genetic analysis in determining mutation-disease associations and may lead to a better understanding of the role of PITX2 in ocular development.

## 1. Introduction

Anterior segment dysgenesis (ASD) encompasses a wide variety of developmental conditions in which multiple tissues, such as the cornea, iris, and lens, are affected [1]. ASD is classified into the following subtypes: Axenfeld-Rieger syndrome (ARS), aniridia, Peters anomaly (PA), and iridogoniodysgenesis. ARS is a rare multisystem autosomal-dominant disorder with a prevalence of approximately 1 in 200,000 [2] and is characterized by complete penetrance but variable expressivity. Ocular phenotypes are mainly characterized by ASD of the eye, such as iris hypoplasia (IH), a prominent Schwalbe line, adhesion of the iris and cornea, corectopia, polycoria, and corneal opacity. The various systemic features observed in ARS patients include facial dysmorphisms, dental anomalies, and redundant periumbilical skin.

ARS can be caused by mutations in the pituitary homeobox 2 (PITX2; OMIM601542) and forkhead box C1 (FOXC1; OMIM601090) genes. Mutations in these two genes are estimated to explain ∼40% of ARS [3]. The PITX2 gene produces three major protein-coding isoforms (PITX2a, PITX2b, and PITX2c), with each containing an identical homeodomain (HD) and C-terminal domain, differing only at the N-terminal region. The common C-terminal domain contains a highly conserved 14-amino acid otp, aristaless, and rax (OAR) domain that mediates PITX2 protein interactions. The human PITX2 mutations identified to date cluster in the HD and C-terminal regions and mainly result in complete or partial loss of function. Dominant-negative and gain-of-function mutations have also been reported but represent an apparent minority [4–6]. Mutations in the PITX2 gene appear more likely to be associated with ocular, dental, and umbilical anomalies, whereas mutations in FOXC1 appear to be associated with isolated ocular or ocular, heart, and/or hearing defects. FOXC1 mutations primarily result in complete or partial loss of function, and duplications of FOXC1 have also been reported.

Aniridia is a rare congenital disorder of either partial or complete hypoplasia of the iris and can be associated with other eye defects, such as corneal opacification, cataract, glaucoma, lens dislocation, ciliary body hypoplasia, foveal hypoplasia, strabismus, and nystagmus [7]. At least 90% of aniridia cases are caused by heterozygous loss-of-function mutations in PAX6 [8]. Rarely, isolated aniridia is caused by mutations in FOXC1 or PITX2.

Mutations in the PAX6, PITX2, and FOXC1 genes have been associated with ARS and aniridia in an autosomal-dominant manner [9–12]. The traits of ARS display wide ranges of variability in severity and manifestations. The severity of ARS phenotypes and the levels of normal PITX2 protein are also correlated [6, 13, 14]. ARS may be misdiagnosed as aniridia or iridocorneal endothelial syndrome (ICE syndrome) based on certain similarities of clinical characteristics. Furthermore, genetic studies and clinical descriptions of Chinese patients with ARS are nearly absent [15–17]. In this manuscript, PITX2 and FOXC1 mutation analysis and detailed clinical evaluations were performed to identify novel mutations and to characterize an unusual ophthalmic phenotype of severe ARS in a Chinese patient.

## 2. Methods

### 2.1. Patients

Twenty-six unrelated patients with different forms of ASD, with or without nonocular defects, were recruited into our study. Anterior segment phenotypes included 5 patients with ARS, 1 patient with a phenocopy of aniridia, and 20 patients with other ASDs (primarily PA); 5 patients had an affected family member (2 with ARS and 3 with other ASDs). Classical aniridia patients with foveal hypoplasia and nystagmus were excluded. Furthermore, 100 normal controls, mainly from Southern China, participated in our study. All participants were enrolled from the Paediatric and Genetic Clinic, Eye Hospital, Zhongshan Ophthalmic Center, Guangzhou, China. Medical and ophthalmic histories were obtained. Ophthalmological examinations were performed by Drs. Guo and Zhang. The anterior and posterior segments of the eyes were documented by slit-lamp examination, specular microscopy, gonioscopy, applanation tonometry, A/B ultrasound scanning, and fundoscopy. Informed consent conforming to the tenets of the Declaration of Helsinki was obtained from each participant prior to the study.

### 2.2. Genetic Analysis

Genomic DNA was extracted from venous blood from each participating individual. Prior to this study, the DNA of these patients had been screened for mutations in PAX6, but none of the tests were positive [18]. All coding regions of PITX2 and FOXC1 were amplified by polymerase chain reaction (PCR) using primers. The primer sequences are listed in Table 1. The primers for exon 4 of PITX2a were adapted from those described by Cella and coworkers [19]. The PCR products were sequenced with an ABI BigDye Terminator cycle sequencing kit version 3.1 (Applied Biosystems, Foster City, CA) according to the manufacturer's recommendations using an ABI sequencer 3100 that was confirmed by our laboratory. For clarity, we studied the PITX2a isoform for mutations in affected patients. Sequencing results from the patients as well as consensus sequences from the NCBI human genome database (PITX2a; NM_153427.1 and FOXC1; NM_001453.2) were imported into the SeqManII program of the Lasergene package (DNASTAR Inc., Madison, Wisconsin) and were aligned to identify variations. Each variation was confirmed by bidirectional sequencing. Mutations were named according to the nomenclature recommended by the Human Genomic Variation Society (HGVS).

### 2.3. Heteroduplex Single-Strand Conformation Polymorphism Analysis

The variations detected in PITX2 and FOXC1 were further evaluated in 100 normal controls by heteroduplex single-strand conformation polymorphism (HA-SSCP) analysis, as previously described [20], using additional pairs of primers (Table 1). Briefly, the PCR product was mixed with an equal volume of gel-loading buffer (95% formamide, 20 mM EDTA, 0.05% bromophenol blue, and 0.05% xylene cyanol), denatured at 95°C for 5 min and immediately placed on ice for 5 min. The DNA samples were loaded directly onto 8% polyacrylamide gels and run for 8 h at 40 W in a solution of 0.5 × TBE at room temperature.

## 3. Results

**Figure 1:**
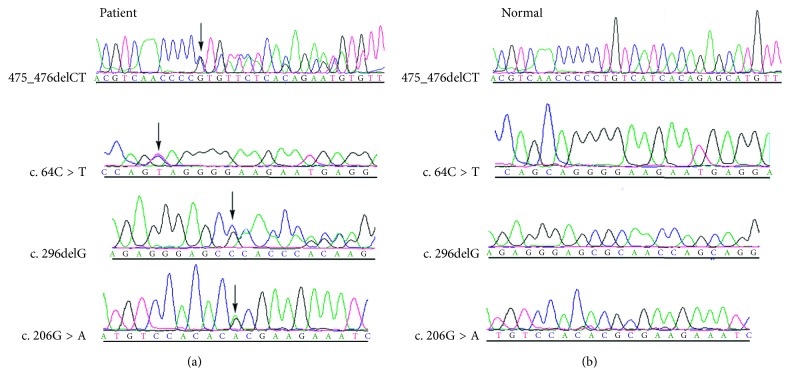
Sequencing analysis of four PITX2-affected patients with ARS. The mutant PITX2 sequence (a) and corresponding normal sequence (b) are shown for patients 1–4. The exact mutation is labelled by an arrow.

**Figure 2:**
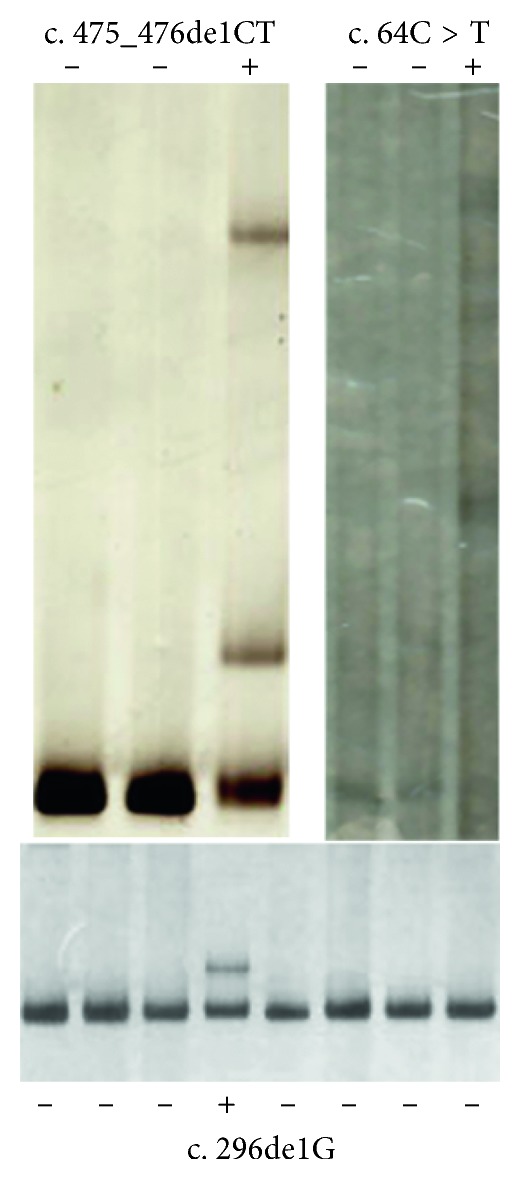
Detection of novel PITX2 mutations by heteroduplex-SSCP analysis. The plus sign (+) indicates samples with heterozygous variation, and the minus sign (−) indicates normal controls.

**Figure 3:**
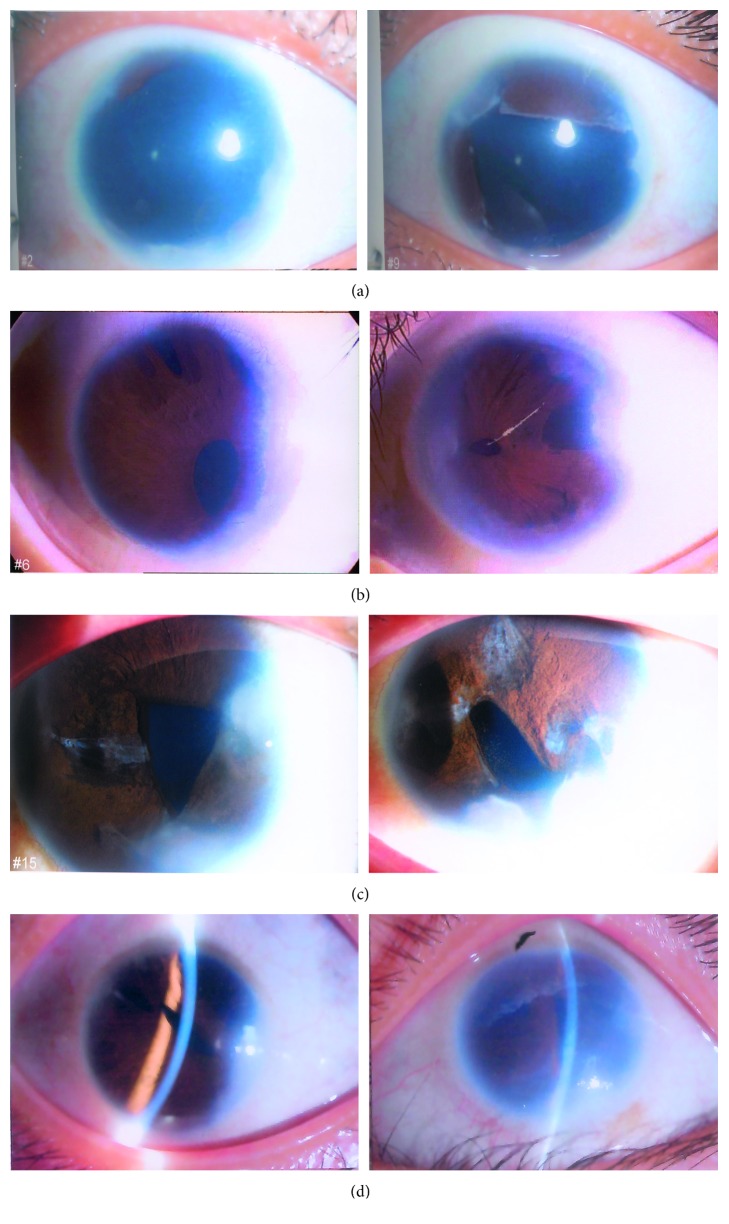
Ocular characteristics of the patients with ARS. (a) Patient 1, in whom both eyes showed megalocornea and complete and partial aniridia and the cornea was centrally opaque, perhaps due to oedema associated with elevated IOP. (b) Patient 2, whose ocular manifestations included microcornea, iris atrophy, and core metamorphosis, iris adherent to cornea, and trabecular meshwork with peripheral corneal opacity in both eyes. (c) Patient 3, who exhibited microcornea, corectopia, core metamorphosis, peripheral corneal opacity in both eyes, and slit pore formation for bilateral iris atrophy. (d) Patient 4, who exhibited microcornea and iris hypoplasia. The pupil was stretched, and corneal oedema was observed in the left eye.

**Figure 4:**
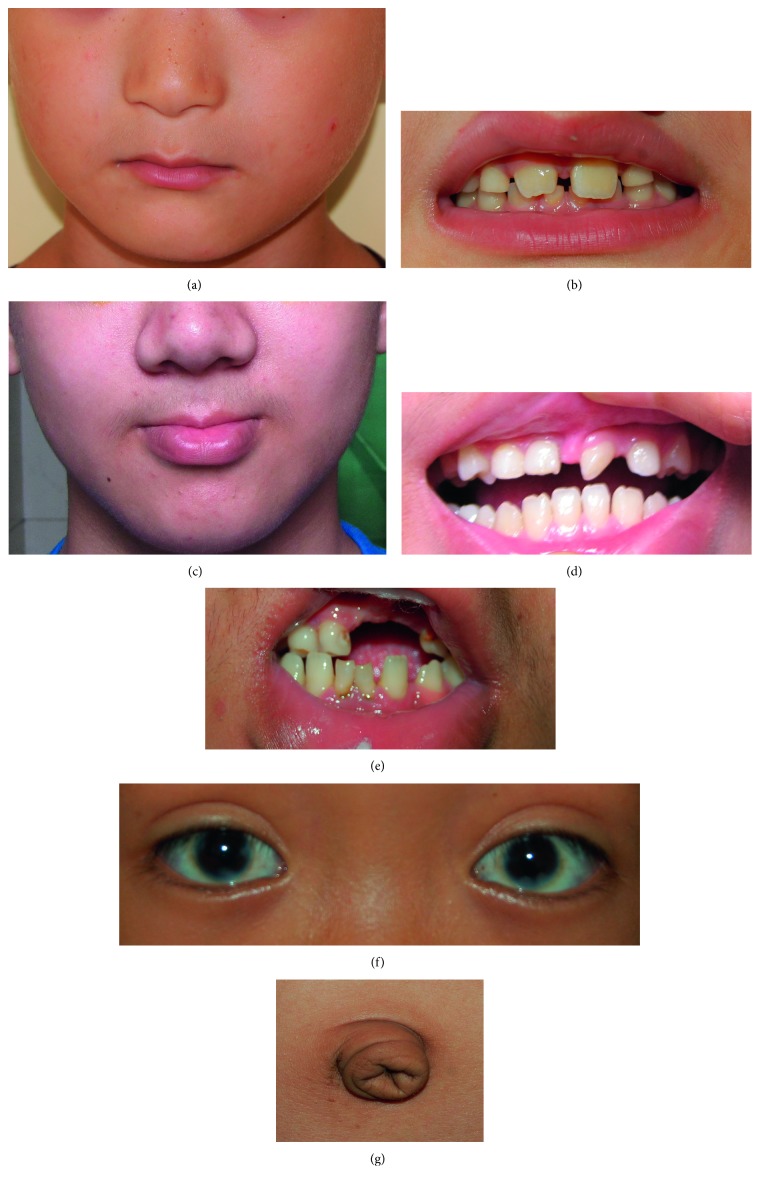
Systemic anomalies of patients with ARS. (a, b) The nonocular manifestations of Patient 1, including midface abnormalities with flattening of the midface, prominent lower lips, and missing and misshapen teeth. (c, d) The systemic features of Patient 2, including flat face, missing and misshapen teeth, and protruding lower lip. (e, f, g) The nonocular manifestations of Patient 3, including hypodontia, flat nasal bridge, telecanthus, and redundant periumbilical skin.

PITX2 mutations were found in four of twenty-six independent patients with ARS (Figure 1). Of the four cases, three were novel. Each variation was confirmed by bidirectional sequencing. None of the novel variations were observed in other relatives or normal controls by HA-SSCP analysis (Figure 2). Examples of the anterior segment findings are shown in Figure 3. The clinical features and mutations in patients with our PITX2 mutations are summarized in Table 2. Systemic anomalies of patients are shown in Figure 4. The four patients had a wide variety of nonocular manifestations, including mild craniofacial dysmorphism, dental anomalies, and redundant periumblical skin, typical for ARS. A total of twenty-six affected individuals were molecularly assessed, and no FOXC1 mutations were detected.

It is of interest that significant differences were noted in the phenotypes of the two eyes of Patient 1. All patients had typical ocular characteristics of Axenfeld-Rieger syndrome in both eyes, except for Patient 1, who presented with complete simulating aniridia in the right eye but partial aniridia in the left eye. Details of the clinical characteristics and evaluation of this patient are reported as follows.

**Figure 5:**
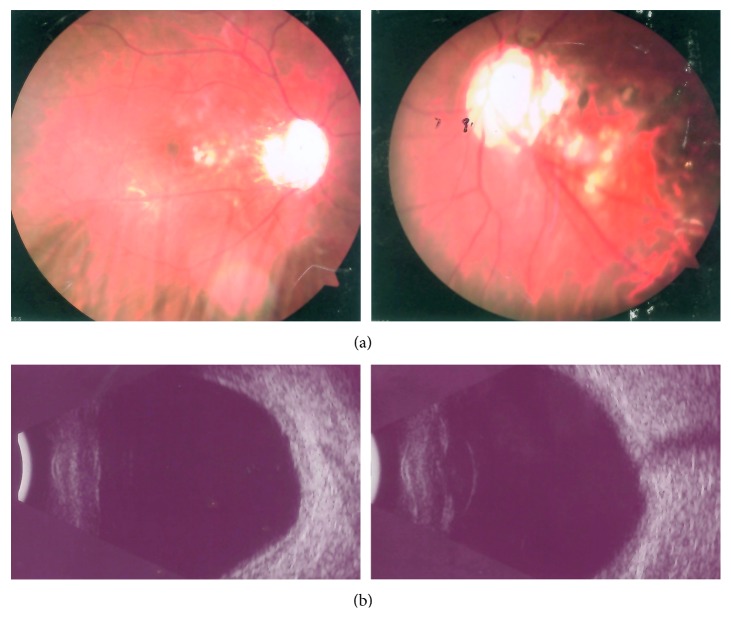
Fundoscopy examination and type-B ultrasonic imaging of Patient 1. The results revealed vitreous opacity and foveal hypoplasia.

Patient 1 (c.475_476delCT; P.L159VfsX39): the patient was a thirteen-year-old female with a high myopia of −6.50*D* in both eyes who presented at three years of age with hazy megalocornea, bilateral posterior polar cataracts, and early-onset severe glaucoma and was diagnosed with aniridia by a local ophthalmologist (Figure 3(a)). The patient's best-corrected visual acuity was counting fingers in both eyes. The cornea was opaque, which was associated with an elevated normal intraocular pressure (IOP) resistant to medication. Endothelial cell counts were 1386 and 1518 in the right and left eyes, respectively. She had posterior embryotoxon in both eyes with broad iris adhesions. Significant findings were noted as the ocular examination displayed different degrees of aniridia in both eyes. The IOPs were 47 mmHg in the right eye and 39 mmHg in the left eye. The anterior chamber appeared extremely shallow with a depth of 1.76 mm in the right eye and 1.91 mm in the left eye. A-scanning demonstrated posterior staphyloma with axial lengths of 25.56 mm in the right eye and 26.06 mm in the left eye. Funduscopy and type-B ultrasound were performed for this patient, and vitreous opacity and foveal hypoplasia were observed. Optic disc examination revealed a cup/disc ratio of 0.90 and showed glaucomatous atrophy of the optic nerve in both eyes (Figures 5(a) and 5(b)). She had hearing defects, missing and misshapen teeth, and midface abnormalities (Figure 4), and foveal hypoplasia was observed, but she did not have nystagmus and, therefore, did not have the classic manifestation features of true aniridia. Because of these findings, she was diagnosed as Axenfeld-Rieger Syndrome. The patient's visual acuity was poor, although trabeculotomies were performed in both eyes. The patient had no recorded family history of ocular disease.

Of the four mutations described, two were frame-shift mutations. The frame-shift mutation c.475_476delCT was predicted to produce a premature stop codon at position 197. A truncated protein with 196 amino acids, including an aberrant 38-amino acid residue, was putatively generated; this protein is 75 amino acids shorter than the wild-type PITX2a protein, which possesses 271 amino acids. Thus, this mutation led to partial loss of the C-terminal domain of PITX2. The c.296delG (P.R99PfsX56) mutation identified in the present study is a novel deletion mutation in exon 6, which causes a frame shift after Arg98 of PITX2a, resulting in a premature stop codon at codon 154, leading to complete loss of the C-terminal domain of PITX2. The nonsense mutation c.64C > T (P.Q22X) resulted in a substitution of glutamine for the stop codon at amino acid position 22, which is located in the N-terminal domain and results in premature termination with complete loss of the HD and C-terminal domain. Hence, the Q22X mutation affects the structure and function of PITX2 and is expected to produce a functionally null allele. The c.206G > A (P.69R > H) mutation, resulting in the replacement of an arginine residue with a histidine at the 69th amino acid position, has been identified in different original patients; this study is the fifth report [21–24]. This mutation appears in different ethnic groups, indicating that c.206G > A may be a hot-spot mutation.

## 4. Discussion

ARS is an autosomal-dominant disorder that can be caused by mutations in the FOXC1 and PITX2 genes. Compared with FOXC1 mutations, PITX2 mutations are more commonly associated with the extraocular systemic abnormalities of ARS. In our case, all PITX2-affected patients had extraocular abnormalities, including redundant periumbilical skin, midface abnormalities, and abnormal dental development. The frequency of FOXC1 gene mutations in patients with ARS ranges from 20% to 30%. No sequence alterations were detected in the FOXC1 gene in any ASD patients. Our results suggest that the PITX2 gene accounts for a significant portion of the Chinese ARS population with additional systemic defects. Such a result is consistent with previous studies in which mutations in the PITX2 gene appeared to be more strongly associated with ARS and extraocular findings.

ARS traits display tremendous variability in severity and manifestation. In this study, of particular note is the frame-shift mutation, c.475_476delCT (P.L159VfsX39), located at the 3′ end of the coding region; this mutation was identified in a Chinese ARS patient. The patient exhibited a phenocopy of bilateral aniridia, in addition to the classic ARS ocular features. This case is the first report of a mutation at the 3′ end of the PITX2-coding region that extends the phenotypic consequences of PITX2 mutations to bilateral aniridia. Glaucoma was diagnosed in patients with our PITX2 mutations; however, the severities were different. Patient 1 was diagnosed with early-onset severe glaucoma when she was three years old. Severe, early-onset glaucoma is typically difficult to control, leading to almost complete optic nerve head damage and more devastating visual loss in both eyes. A correlation between the severity of ARS phenotypes and normal PITX2 protein levels was also noted. In the literature, only three cases of ARS with features of bilateral or unilateral aniridia with PITX2 mutations have been found. One case was affected by a splicing mutation that introduced a premature termination, and two other cases were affected by nonsense mutations. In 2000, Rahat Perveen first described a phenocopy of bilateral aniridia carrying a G-C 3′ splice site of intron 2; the patient had best-corrected visual acuities of 6/9 and 6/12 [25]. Véronique Vieira and coworkers identified an E55X nonsense mutation in an ARS patient who presented with complete aniridia associated with goniostrands in the right eye and major corectopia associated with partial aniridia in the left eye [26]. In 2012, Law et al. reported an asymmetric phenotype of the anterior segment with features of Axenfeld-Rieger anomaly in one eye, but aniridia in the other eye. A nonsense mutation, p.Q21X for PITX2a or p.Q67X for PITX2b, was found in patient M.A, who had best-corrected visual acuities of 20/80 in the right eye and 20/40 in the left eye [27]. PITX2a is a 33 kDa homeodomain protein. The HD is responsible for recognizing specific DNA sequences to bring transcription factors to the appropriate target genes. The integrity of PITX2 is essential for binding DNA and is critical for PITX2 to act as a transcription factor [28]. All of the above-reported mutations were predicted to abolish the DNA-binding functions of the affected allele and to lead to a premature termination codon (PTC) and are subject to nonsense-mediated mRNA decay (NMD). The mechanism for the PITX2-related severity of the ocular phenotype in these patients may be the consequence of PITX2 haploinsufficiency. These mutations lead to severe aniridia-emulating phenotypes but result in a relatively good visual acuity. In this study, the P.L159VfsX39 mutation is a novel deletion mutation at the 3′ end of the PITX2-coding region, encoding a normal HD but partially truncating the PITX2 C-terminus, which causes a frame shift after Pro158 of PITX2a and introduces a PTC in exon 6. As this PTC is located in the final exon of the gene, it is predicted to escape NMD. Many researchers have noted that aniridia phenotypes are caused by mutations that introduce a PTC; however, they have also suggested that 3′ mutations, which introduce a PTC into the PAX6 open reading frame, do in fact yield dominant-negative alleles that may cause more severe phenotypes [29, 30]. Until now, there have been only 2 reports of PITX2 dominant-negative mutations that have been characterized at the cellular level [5, 14, 31]. Saadi et al. have noted that lysine at residue 50 in the PITX2 HD plays important roles in both DNA-binding and dimerization activities. The authors also suggested that dimerization appears to involve both the C-terminal region and the HD, as exemplified by the dominant-negative PITX2 K88E protein [4]. Unlike previously reported PITX2-associated aniridia mutations, this P.L159VfsX39 frame-shift mutation retained the position 50 residue of this HD. Therefore, we predicted that the truncating mutation at the 3′ end of PITX2, P.L159VfsX39, escapes NMD and generates dominant-negative proteins that cause much more severe phenotypes than the abovementioned PITX2 mutations. The PITX2 C-terminus has both inhibitory and stimulatory activities [4]. Irfan Saadi and colleagues reported that the PITX2 mutations at the 3′ part of the coding region, W133Stop and D122FS, may result in gain-of-function phenotypes in ARS patients at the cellular level [4, 32]. One caveat of this interpretation is that the mutants may not be expressed in patients because of nonsense-mediated mRNA decay or protein degradation. These results indicate the complexity and potential ramifications of mutations in the PITX2 C-terminal tail in ARS patients. Hopefully, additional research in this area may eventually clarify the exact mechanism of these mutations.

**Figure 6:**
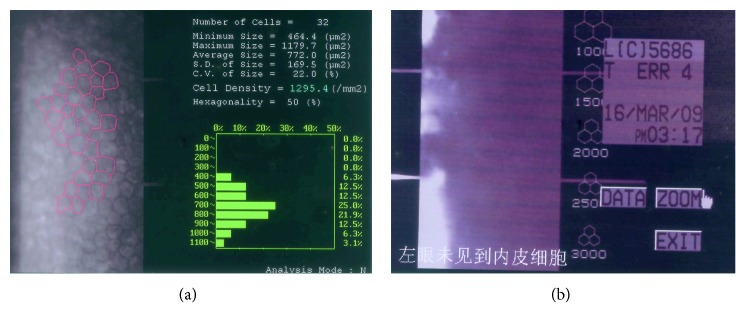
Specular microscopy of Patient 4. (a) Reduced endothelial densities but normal morphology in size and shape in the right eye. (b) The corneal stroma were centrally opaque and associated with the permeability of the endothelial barrier in the left eye.

The iris manifestations in ASDs are important in making a diagnosis. Clinically, ARS may be confused with ICE syndrome or aniridia on the basis of clinical similarities. In our case, for instance, Patient 1 was originally diagnosed with aniridia, and foveal hypoplasia was observed, but she did not have nystagmus and, therefore, did not have the classic manifestations of true aniridia. Patient 4 was misdiagnosed with ICE syndrome; specular microscopy showed reduced endothelial densities but normal morphology, which is inconsistent with ICE syndrome (Figure 6). For patients presenting with IH and glaucoma, careful evaluations of endothelial morphology, iris, fundus, foveal development, and extraocular manifestations are critical.

In summary, we expanded the mutation spectrum of the PITX2 gene in individuals with ARS. Our results suggest that the PITX2 gene accounts for a significant portion of the Chinese ARS population with systemic abnormalities. This study is the first reported case of a mutation at the 3′ end of the PITX2-coding region that extends the phenotypic consequences of PITX2 mutations to bilateral aniridia. Therefore, we speculated that ARS traits can display tremendous variability in severity and manifestation for the PITX2 dominant-negative effect. These results further emphasize the importance of careful clinical and genetic analysis and lead to a better understanding of the role of PITX2 in the normal development of the eye.

## Figures and Tables

**Table 1:** Primers for amplifying and sequencing PITX2 and FOXC1.

Gene	Exon	Forward primer (5′-3′)	Reverse primer (5′-3′)	Product size (bp)	Annealing temperature (°C)
PITX2	2	GGCCGCCGCTTCTTACA	CACTGGCGATTTGGTTCTGATTT	254	63
	3	CTGCCTCGCCCCTCCCCCTCCTC	TTCAAGCAGCAGGCGCGTCAGGTC	350	64
	4	GGGGCAGTAGCCAAGGACT	CAGCTAAGCGGGAATGTCTG	289	64
	5	GGCGCGGACACCCACACTTG	GCGCTGCCTTCCACATTCTCT	487	65
	6-A	GCGCTGCCTTCCACATTCTCT	GGTGGATAGGGAGGCGGATGTAAG	346	63
	6-B	GGTGGATAGGGAGGCGGATGTAAG	CGACGGGCTACTCAGGTTGTTCA	256	64
	6-C	TCCCGGGCTCCAGTCTCAACAG	TTTCTTTAGTGCCCACGACCTTCT	355	64
	SSCP-1	CGACGACATGTACCCAGGCTATTC	GCGACGGGCTACTCAGGTTGTT	257	68
	SSCP-2	GGCGCGGACACCCACACTTG	CTGCCGCCTTTGCCGCTTCTTCTT	269	68
	SSCP-3	CTGGCCCTGGTATCTTGGTGTGC	GGTGGATAGGGAGGCGGATGTAAG	346	63

FOXC1	1-A	CCCGGACTCGGACTCGGC	AAGCGGTCCATGATGAACTGG	429	63
	1-B	GCGCACGCCGAGCAGTA	CACCGCGTCCTTCTTCTTGA	390	60
	1-C	CACCCTGAACGGCATCTACCA	AGGCTGCTGCTGCTGCTGTCG	504	64
	1-D	GCCCGTGCGCATCCAGGACATCAA	GCTGCCCGCGCTGGAGGTCTGG	463	64
	1-E	AGGGCTTCAGCGTGGACAACATCA	GGTGGGCCGCAGGGTGGTG	482	65
	1-F	CAAGCCATGAGCCTGTACG	GGGTTCGATTTAGTTCGGCT	502	60

Primer sequences, sizes of PCR products, and annealing temperatures used for the amplification are listed. Primers 2–6 were used to amplify and sequence the PITX2-coding segments. The sequencing of exon 6 of PITX2 was performed with three overlapping primers A–C. Primers SSCP-1 to SSCP-3 were used to amplify the novel variations detected in PITX2 for heteroduplex-SSCP analysis. The single FOXC1 exon was sequenced with six overlapping primers, A–F.

**Table 2:** Summary of clinical findings and mutations in patients with PITX2 mutations.

Patient	Diagnosis	Mutations (isoform a)	Age of onset (year)	IOP (mmHg) (OD; OS)	BCVA (OD; OS)	Corneal changes (OU)	Ocular findings (OU)	Dental anomalies	Other findings
1	ARS glaucoma cataract high myopia	c.475_476delCT (P.L159VfsX39)	3	47; 39	FC10; FC50cm	Megalocornea	Aniridia, PE	Missing and misshapen teeth	Midface abnormalities hearing defects
2	ARS glaucoma	c.64C > T (P.Q22X)	7	26; 29	0.2; 0.1	Microcornea peripheral corneal opacity	Iridocorneal adhesions coremetamorphosis, corectopia, IH	Missing and misshapen teeth	Flat face prominent lower lips
3	ARS glaucoma	c.296delG (P.R99PfsX56)	11	25; 44	0.5; 0.3	Peripheral corneal opacity	Iridocorneal adhesions coremetamorphosis, corectopia, IH	Hypodontia	Redundant periumbilical skin telecanthus, flat nasal bridge
4	ARS glaucoma	c.206G > A	11	29; 41	0.6; 0.05	Microcornea	Stretched pupil	Microdontia	Midface abnormalities, appendages

Clinical characteristics, including age of onset, IOP, BCVA, corneal changes, ocular findings, dental anomalies, and other findings, are shown in this table. Abbreviations: IOP, normal intraocular pressure; BCVA, best-corrected visual acuity; PE, posterior embryotoxon; IH, iris hypoplasia; FC, finger count.

## Data Availability

The data used to support the findings of this study are restricted in order to protect patient privacy. The data supporting the conclusions of the study are available from Liqin Huang on request.
